# Systems Level Analysis and Identification of Pathways and Networks Associated with Liver Fibrosis

**DOI:** 10.1371/journal.pone.0112193

**Published:** 2014-11-07

**Authors:** Mohamed Diwan M. AbdulHameed, Gregory J. Tawa, Kamal Kumar, Danielle L. Ippolito, John A. Lewis, Jonathan D. Stallings, Anders Wallqvist

**Affiliations:** 1 Department of Defense Biotechnology High Performance Computing Software Applications Institute, Telemedicine and Advanced Technology Research Center, U.S. Army Medical Research and Materiel Command, Fort Detrick, Maryland, United States of America; 2 U.S. Army Center for Environmental Health Research, Fort Detrick, MD, United States of America; Michigan State University, United States of America

## Abstract

Toxic liver injury causes necrosis and fibrosis, which may lead to cirrhosis and liver failure. Despite recent progress in understanding the mechanism of liver fibrosis, our knowledge of the molecular-level details of this disease is still incomplete. The elucidation of networks and pathways associated with liver fibrosis can provide insight into the underlying molecular mechanisms of the disease, as well as identify potential diagnostic or prognostic biomarkers. Towards this end, we analyzed rat gene expression data from a range of chemical exposures that produced observable periportal liver fibrosis as documented in DrugMatrix, a publicly available toxicogenomics database. We identified genes relevant to liver fibrosis using standard differential expression and co-expression analyses, and then used these genes in pathway enrichment and protein-protein interaction (PPI) network analyses. We identified a PPI network module associated with liver fibrosis that includes known liver fibrosis-relevant genes, such as tissue inhibitor of metalloproteinase-1, galectin-3, connective tissue growth factor, and lipocalin-2. We also identified several new genes, such as perilipin-3, legumain, and myocilin, which were associated with liver fibrosis. We further analyzed the expression pattern of the genes in the PPI network module across a wide range of 640 chemical exposure conditions in DrugMatrix and identified early indications of liver fibrosis for carbon tetrachloride and lipopolysaccharide exposures. Although it is well known that carbon tetrachloride and lipopolysaccharide can cause liver fibrosis, our network analysis was able to link these compounds to potential fibrotic damage before histopathological changes associated with liver fibrosis appeared. These results demonstrated that our approach is capable of identifying early-stage indicators of liver fibrosis and underscore its potential to aid in predictive toxicity, biomarker identification, and to generally identify disease-relevant pathways.

## Introduction

Exposure to toxic chemicals can lead to liver injury through a variety of mechanisms, such as oxidative stress, the immune response, activation of apoptotic pathways, and necrosis [1]. Liver fibrosis is a common pathologic feature observed in a wide spectrum of liver injuries [2], [3] and is marked by inflammation and excessive accumulation of extracellular matrix (ECM) components [4]. Liver fibrosis results in scar formation and, if unresolved, leads to cirrhosis, portal hypertension, and liver failure [4]. Liver fibrosis typically starts with apoptosis or necrosis of hepatocytes, which causes reactive oxygen species generation. This process leads to the release of inflammatory mediators and ultimately results in activation of hepatic stellate cells [3], the main ECM-producing cells in the liver. This activation of hepatic stellate cells is the key pathogenic mechanism of liver fibrosis [3]–[6]. Activated hepatic stellate cells lead to further inflammation and ECM generation, which results in the replacement of liver parenchymal cells with ECM [5]. Despite recent progress, our understanding of the molecular mediators of liver fibrosis remains incomplete, and we are still in the process of identifying such mediators [7], [8].

Although fibrotic damage is reversible, there are no approved drugs or treatments for liver fibrosis. Key in understanding damage and control of fibrosis is accurate diagnosis or early indicators of damage. The gold standard for diagnosing fibrosis is currently via liver biopsy. This invasive method has many limitations, such as inter- and intra-observer variations and sampling variability [9]. Thus, there is a need to identify sensitive, specific, and non-invasive biomarkers of liver fibrosis. Identification of such biomarkers will improve diagnosis and allow better clinical management of the disease. In the military, this capability would aid in field assessment and potentially enable timely evacuation or guide return-to-duty decisions. Elucidation of the pathways and networks associated with liver fibrosis will provide insight into the molecular mechanisms of this disease and, importantly, help us to identify mechanism-based biomarkers of liver damage.

Computational systems biology approaches are now routinely used to analyze gene expression data and to gain insight into the molecular mechanisms of many diseases [10]–[15]. Pathway enrichment analysis provides a biological interpretation of gene lists obtained from microarray data using manually curated pathway databases, such as the Kyoto Encyclopedia of Genes and Genomes (KEGG) and Reactome [16], [17]. The BioSystems database [18], [19] provides an integrated resource of pathways from several major pathway databases, including KEGG and Reactome. Huang et al. [20] have summarized the various tools and statistical methods available for pathway enrichment analysis and their utility in elucidating the mechanisms of diseases [21]–[23]. In literature related to liver fibrosis, the work of Affo et al. [24] utilizes KEGG pathway analysis in identifying the role of focal adhesion and cytokine-cytokine receptor interaction pathways in alcoholic hepatitis. Although widely used, pathway analysis has some limitations [25]. Foremost, pathway analysis relies exclusively on experimentally confirmed, curated data. Only a small fraction of human genes map to curated pathway collections (e.g., KEGG [14]); thus, pathway analysis is inherently biased against the discovery of new molecular mediators. For example, we found that only 5,870 of the ∼21,000 human genes mapped to the 229 KEGG pathways [26]. Moreover, the gene networks comprising the pathway maps are not mutually exclusive, and the same gene can occur in many pathways [25]. Integrated analyses of gene expression data with protein-protein interaction (PPI) networks enable us to partly overcome the limitations associated with pathway analysis. The potential of this integrated approach has been shown in identifying biomarkers for breast cancer [27] and in understanding the molecular mechanisms of dilated cardiomyopathy [28], hepatitis C virus infection [29], and cancer and heart disease [30]–[34]. However, to the best of our knowledge, no such integrated analysis has been reported for liver fibrosis.

Thus, our goal in this study was to identify liver fibrosis-relevant pathways and networks based on an integrated gene expression and PPI network analysis. We analyzed the gene expression data from a range of chemical exposure conditions that produced periportal liver fibrosis in DrugMatrix, a publicly available toxicogenomics database [35]. We carried out differential expression and co-expression analysis using rank product and hierarchical clustering, respectively, to identify genes associated with liver fibrosis [36]. We then examined these genes in two separate analyses. In the first analysis, we identified the KEGG pathways associated with liver fibrosis. In the second analysis, we integrated the gene expression data with the high-confidence human PPI network to obtain liver fibrosis-relevant sub-networks [37]. We identified a PPI network module associated with liver fibrosis that includes known liver fibrosis-relevant genes like *Timp1*, *Lgals3*, *Ctgf*, and *Lcn2*, along with several new genes. We further analyzed the expression pattern of the genes in the PPI network across a wide range of 640 chemical exposure conditions in DrugMatrix and linked compounds such as carbon tetrachloride to potential fibrotic damage before histopathological evidence of liver fibrosis appeared. These results illustrate the potential of our approach to aid in toxicity prediction and biomarker discovery.

## Materials and Methods

**Figure 1:**
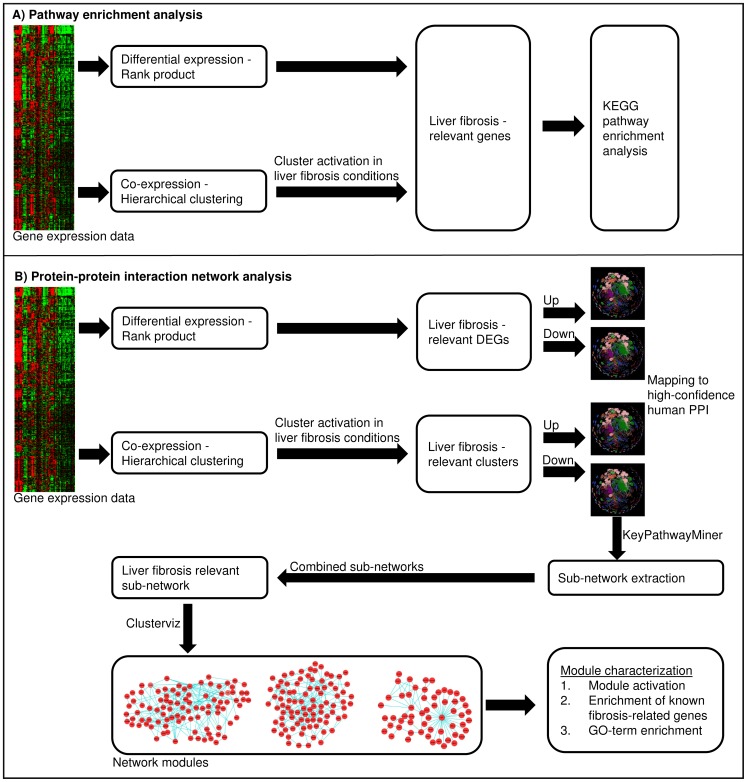
Workflow used in this study to identify pathways and networks associated with liver fibrosis.

**Figure 1** shows the overall workflows used in this study to identify liver fibrosis-relevant pathways and interaction networks. We used two separate but complementary approaches to map the overall gene transcriptional response to liver fibrosis via *1*) enrichment analysis of knowledge-based pathways association, and *2*) integration of gene expression data with PPI networks to identify interaction networks. These fibrosis-relevant interaction networks can be considered as *de novo* pathways.

### Data collection and processing

We used DrugMatrix, a publicly available toxicogenomics database [35], [38], for our analyses. DrugMatrix is a large collection of gene expression, hematology, histopathology, and clinical chemistry data obtained from Sprague-Dawley rats after exposure to a range of chemicals, including industrial chemicals, toxicants, and drugs with multiple time intervals, dose ranges, and tissues for each chemical [35]. A chemical exposure condition in DrugMatrix data refers to exposure to a particular chemical at a particular dose and time. We downloaded the DrugMatrix liver gene expression data generated using Affymetrix GeneChip Rat Genome 230 2.0 Arrays from the National Institute of Environmental Health Sciences (NIEHS) server (https://ntp.niehs.nih.gov/drugmatrix/index.html). Of the 2,218 microarray CEL files in this database, 1,941 were chemical exposures and 277 were controls. We performed background correction, quantile normalization, and summarization using the robust multi-array average method in R/BioConductor package *affy* [26], [39]–[41]. We then used the BioConductor package *ArrayQualityMetrics* to assess the quality of the microarray data and removed 155 outlier arrays [42]. We reprocessed the remaining arrays using the robust multi-array average method and used this final normalized data for all our analyses. With the BioConductor package *genefilter*, we carried out non-specific filtering of the genes [43]. We removed probe sets without Entrez ID or with low variance across chemical exposures based on inter-quartile range. We additionally filtered probe sets based on “Present” calls using the BioConductor package *affy*, retaining only the probe sets for which at least 25% of the chemical exposures had “Present” calls for all replicates within the chemical exposure condition. After calculating the average intensity between the replicates of a chemical exposure condition, we computed log-ratios for each gene between treatments and their corresponding controls. Our final log-ratio matrix contained 7,826 genes and 640 chemical exposure conditions.

### Identifying genes relevant to liver fibrosis

The DrugMatrix database provides histopathology data associated with each chemical exposure condition. There were five chemical exposure conditions that produced liver periportal fibrosis with a histopathology score >1 (**Figure S1**). We carried out a quality check by clustering the replicates of these five chemical exposures, along with their respective controls. Ideally, the chemical exposures and controls would have clustered separately. But all replicates of 5-day exposures to Crotamiton-750 mg/kg clustered with controls rather than with other treatments (**Figure S1**). Hence, we excluded this condition from the liver fibrosis-producing condition set. **Table 1** lists the four chemical exposure conditions that were used in this study as the liver fibrosis-producing condition set. All replicates of the four chemical exposure conditions had a histopathology score of 2. We used the rank product method to identify differentially expressed genes (DEG) [36]. Rank product is a non-parametric, permutation-based method that has been widely used in many studies [36], [44], [45]. With this method, the fold-change values were converted into rank, and then the significance of the obtained rank, including the false discovery rate (FDR) *p*-value, was calculated. We used the BioConductor package *RankProd* for this analysis [44]. This method produces separate lists of up-regulated and down-regulated genes. We considered all genes with an FDR <0.05 to be significantly differentially expressed. We carried out the rank product analysis separately for each of the four chemical exposure conditions; the genes that were significantly differentially expressed in at least two of the four chemical exposure conditions that produced periportal liver fibrosis were considered as fibrosis-relevant DEGs.

**Table 1:** Chemical exposure conditions that produced periportal liver fibrosis.

Chemical	Dose (mg/kg)	Duration (days)	Histopathology (severity score)
1-Naphthylisothiocyanate	30	7	2
1-Naphthylisothiocyanate	60	7	2
4,4′-Methylenedianiline	81	5	2
N-Nitrosodimethylamine	10	5	2

We carried out hierarchical clustering using the R package *hclust* to identify co-expressed genes [21]. We clustered the genes in the log-ratio matrix using their log_2_ ratio values across 640 chemical exposure conditions. We used the Pearson correlation and the average linkage method to perform the clustering, and the R package *dynamicTreeCut* for automated extraction of clusters [46]. The dynamic tree cut algorithm implements an automated iterative process to identify and split sub-clusters from a dendrogram until the minimum cluster size threshold is reached [46]. We used the *cutreeDynamic* function in this package with *minimum cluster size* set to *16, method* set to *hybrid*, and *deepsplit* set to *True*.

We calculated cluster activation scores to identify liver fibrosis-relevant clusters in order to identify clusters whose constituent genes show altered gene expression (either up- or down-regulation) in chemical exposure conditions that produce periportal liver fibrosis. To calculate a cluster activation score, we first normalized the log-ratio values of each gene across 640 chemical exposure conditions by converting them into Z-scores. The Z-score of gene *i* under chemical exposure condition *j* is given by
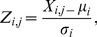
(1)where 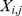 is the log-ratio value for gene *i* under chemical exposure condition *j*, 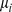 is the average log ratio for gene *i* across all 640 chemical exposure conditions, and 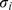 is the standard deviation of the log ratio for gene *i* across all 640 chemical exposure conditions. Next, we obtained the cluster activation scores for liver fibrosis by averaging the Z-scores of all genes within a cluster and across all chemical exposure conditions that produced periportal liver fibrosis. The activation score 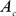 of cluster *c* in liver fibrosis is given by
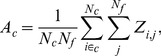
(2)where 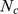 is the number of genes associated with cluster *c*, 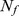 is the number of chemical exposure conditions that produce periportal liver fibrosis, and 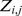 is the Z-score of gene *i* under chemical exposure conditions that produce periportal liver fibrosis *j*. We used an absolute cluster activation score cutoff of 2, corresponding to the 95^th^ percentile of the probability density distribution, and selected genes in clusters above this threshold as liver fibrosis-relevant co-expressed genes.

In general, to find disease-relevant genes, either differential expression or co-expression analysis is commonly used. Utilization of both approaches together will allow us to overcome the limitation associated with each approach. Hence, we combined the gene list from both approaches, i.e., differential expression and co-expression, to form the liver fibrosis-relevant gene set.

### Pathway enrichment analysis

We used the Database for Annotation, Visualization, and Integrated Discovery (DAVID) tool to perform KEGG pathway enrichment analysis [47]. The pathways below a Benjamini-Hochberg FDR <0.05 were considered to be significantly enriched. We used the liver fibrosis-relevant gene set, i.e., the combined list of genes determined by differential expression and co-expression analysis to be relevant to liver fibrosis, for the pathway enrichment analysis. Then we separately used the up-regulated genes and down-regulated genes in this set and carried out pathway enrichment analysis.

### PPI network analysis

Yu et al. [37] used the interaction detection based on shuffling (IDBOS) approach to generate a high-confidence PPI network and showed that the resulting high-confidence PPIs reduce the noise inherent in aggregated PPIs. In their work, they created experimentally derived high-confidence PPI networks for both rats and humans [37]. The rat high-confidence PPI network contained 1,001 nodes, whereas the human high-confidence PPI network consisted of 14,230 nodes. We chose to use the human high-confidence PPI network due to its much larger coverage. The rat probe-set identifiers were mapped to their corresponding human gene identifiers using orthology mapping tools [26], [48], [49]. This approach followed the work by Zhang et al. [50] for mapping other species' gene expression data to a human PPI network. We utilized the KeyPathwayMiner program in Cytoscape 2.8 to obtain the liver fibrosis-relevant sub-network [51]–[53]. KeyPathwayMiner attempts to find maximally connected sub-networks for the input query genes with gene expression data using the ant-colony optimization algorithm [51]. We used KeyPathwayMiner with *ant-colony optimization algorithm*, *node exceptions (K)* set to *100*, and *case exceptions (L)* set to *0*. The *node exception (K)* value provided the allowance for genes that were not present in the input gene set and the *case exception (L)* defined the number of conditions in which the input gene may not be active. We separately ran KeyPathwayMiner using the up-regulated and down-regulated genes in liver fibrosis-relevant DEGs and co-expressed genes and extracted the sub-networks. We then combined the four sub-networks into one final liver fibrosis-relevant sub-network. This sub-network was created by the union of the four sub-networks. We did not use the intersection of the four sub-networks, as it contained only two nodes. Finally, we clustered this liver fibrosis-relevant sub-network using the topology-based network clustering program in Cytoscape, Clusterviz. We used the *EAGLE algorithm* in Clusterviz with default parameters [54]. As implemented, Clusterviz generated 11 network modules.

### Module characterization

We mapped the proteins in the PPI network modules to the rat gene expression Z-score matrix. We calculated the activation scores (**Equation 2**) for the 11 network modules under conditions that caused periportal liver fibrosis. The method is the same as described above, except that here we used the genes in the module instead of cluster. We collected 28 known liver fibrosis-relevant genes from literature (*Set 1*) [3], [5], [55], [56]. Of these 28 genes, 26 mapped to the high-confidence human PPI network. We also collected genes that are known to be associated with liver cirrhosis from the Comparative Toxicogenomics Database (CTD) (*Set 2*) [57]. Of the 126 genes with direct evidence of an association with liver cirrhosis, 95 mapped to the high-confidence human PPI network. We used the Fisher exact test to calculate the enrichment of these genes (*Set 1* and *Set 2*) in each PPI network module. The module genes were also characterized by gene ontology (GO) biological process-term enrichment using the DAVID tool. We used the Revigo tool to visualize the GO enrichment results [58] and analyzed the network modules in terms of activation in liver fibrosis, enrichment with known liver fibrosis-relevant genes, and enrichment of liver fibrosis-relevant GO terms. Based on this analysis, we prioritized one PPI network module (M5) as a liver fibrosis-relevant network module.

We used two statistical significance tests to analyze whether the network module M5 was obtained by random chance. Our null hypothesis was that the observed number of nodes (M5_nodes_) and edges (M5_edges_) in module M5 were obtained by random chance. In the first analysis, we randomly selected 92 proteins from the human PPI network and counted the number of nodes (R_nodes_) and edges (R_edges_) of the largest connected component. This process was repeated 1,000 times. We computed the number of times R_nodes_≥M5_nodes_, denoted as N_randnode_. Similarly, we computed the number of times R_edges_≥M5_edges_, denoted as N_randedge_. Then we computed the probability of obtaining a similar number of nodes by random chance using P  =  N_randnode/_1,000, and the probability of obtaining a similar number of edges by random chance using P  =  N_randedge/_1,000. In the second analysis, we shuffled the human PPI network and then mapped the proteins in the M5 network to this randomized network. We preserved the average node degree during network shuffling. Similar to the first analysis, we extracted the largest connected component, counted the number of nodes and edges, and calculated the probability of obtaining M5 parameters by random chance. We analyzed the overall robustness of the M5 module by comparing the modules generated from a reduced number of samples to those generated from the full dataset. We left out one quarter of the samples from the differential gene expression dataset and analyzed the remaining samples, repeating this procedure four times and leaving out each quarter of the data once. We then compared the overlap of the final module proteins from these four analyses with the module M5 proteins and found an average overlap of 72%. This showed that our method identified roughly the same genes even when samples were missing. We analyzed the expression of genes in module M5 in chemical exposures that produced periportal liver fibrosis across different time periods of exposures. Among the four chemical exposure conditions that produced periportal liver fibrosis, earlier time points were not available for exposure with N-nitroso dimethylamine at 10 mg/kg. Data were available for exposures to 1-naphthyl isothiocyanate at 30 mg/kg and 60 mg/kg at all time points, i.e., 0.25 day, 1 day, and 3 days, and for exposures to 4,4'-methylene dianiline at 1 day and 3 days. We mapped the expression profile of genes in module M5 across different time periods using the average log_2_ ratio of the available chemical exposure data at that time point. Finally, the genes in the prioritized network module M5 were used to cluster the 640 chemical exposure conditions in the DrugMatrix database. We used the clustering software *cluster3* for this purpose [59]. We evaluated the specificity of network module M5 using the average Z-score across the genes in M5 for each of the 640 chemical exposure conditions.

### External validation

In order to further demonstrate the relevance of network module M5 in liver fibrosis, we evaluated the M5 genes in two external datasets (GSE13747 and GSE6929) from the Gene Expression Omnibus (GEO). Both datasets used Affymetrix GeneChip Rat Genome 230 2.0 Arrays. In GSE13747, liver fibrosis was produced by bile duct ligation [60]. Six replicates of liver fibrosis samples and six controls were available. In GSE6929, liver cirrhosis was induced by inhalation of carbon tetrachloride [13]. Four replicates of liver cirrhosis controls and four replicates of sunitinib (SU11248)-treated samples were available. We used the same steps described above for the DrugMatrix database to preprocess these two external datasets and calculated the log_2_ ratio between the treatment and controls. Finally, we matched the genes in module M5 and calculated the correlation between the average log_2_ ratios in the four chemical exposure conditions that produced periportal liver fibrosis from the DrugMatrix database with the log_2_ ratio from external datasets.

## Results and Discussion

### Identification of liver fibrosis-relevant genes

**Figure 2:**
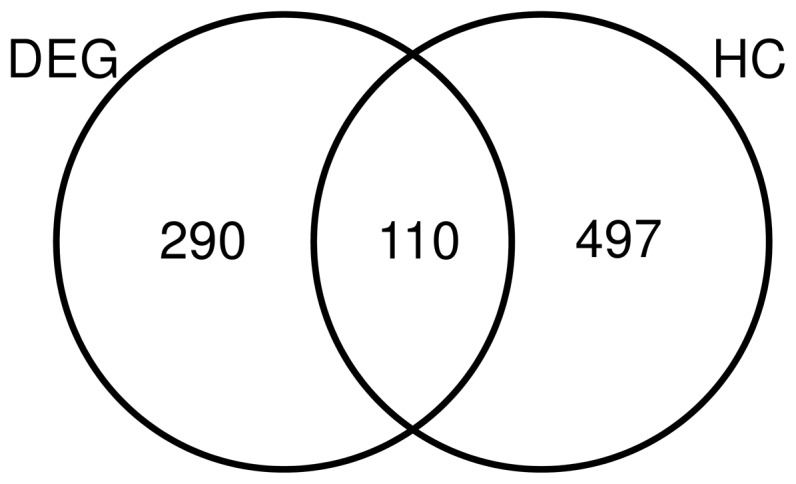
Number of fibrosis-relevant genes from differential and co-expression analysis. Number of genes in the liver fibrosis-relevant differentially expressed gene list and liver fibrosis-relevant co-expressed gene list and the overlap between them.

We used differential gene expression and co-expression analysis to identify liver fibrosis-relevant genes. As outlined in the Materials and Methods section, we analyzed the chemical exposures in the DrugMatrix data that produced periportal liver fibrosis with a histopathological score >1. We used the rank product approach and identified 400 liver fibrosis-relevant DEGs, of which 192 genes were significantly up-regulated, and 208 genes were significantly down-regulated. Here, we used an FDR <0.05 as the cutoff value to select DEGs. This cutoff-based approach knowingly excludes DEGs to minimize false positives and may not capture the complete picture of the disease or processes being studied [61]–[63]. For example, many genes that do not meet the cutoff criteria can be involved in the same pathway as the DEGs and provide insights into the altered disease process [63]. Identifying co-expressed genes by means of gene clustering is an alternative approach that does not use cutoffs or thresholds at the individual gene level. Instead, genes are clustered based on their expression profiles across a wide range of exposures in order to identify gene sets that are expected to have similar functions, i.e., participate in related pathways [64]. We used hierarchical clustering to cluster 7,826 genes based on their log-ratio values across 640 chemical exposure conditions, which yielded 210 gene clusters containing an average of 37 genes each. Unlike differential expression analysis, these co-expressed genes were not linked or associated with liver fibrosis or any other particular disease. We used the cluster activation scores defined in **Equation 2** to establish the connection between the gene clusters and liver fibrosis. We found 565 genes in the nine clusters with activation scores >2 and 42 genes in the two clusters with activation scores <−2. The genes in these clusters were used as liver fibrosis-relevant co-expressed genes. Finally, we combined the differentially expressed and co-expressed gene lists to create a liver fibrosis-relevant gene set with 897 genes. There were 110 genes in common between the differentially expressed and co-expressed genes, and we show the overlap between these two sets as a Venn diagram (**Figure 2**). **Table S1** provides the list of 897 genes, along with log-ratio values, in the four chemical exposure conditions that produced periportal liver fibrosis.

### Pathway enrichment analysis

**Table 2** lists the significantly enriched KEGG pathways derived from the liver fibrosis-relevant gene set. These pathways include leukocyte transendothelial migration, focal adhesion, chemokine signaling, regulation of the actin cytoskeleton pathway, and ECM-receptor interaction, and they mainly represent liver fibrosis-related processes. These processes are consistent with previous reports for liver fibrosis. Injured liver cells and activated hepatic stellate cells release chemokines that recruit leukocytes to the site of injury [65]. An elevated expression of chemokines and chemokine receptors has been reported in both animal models and clinical cases of liver fibrosis [66]. Hepatic stellate cell migration is an essential process in fibrosis [67]. Hepatic stellate cells are also known to express adhesion molecules and α-smooth muscle actin (SMA) [4]. Activated hepatic stellate cells are known to secrete prion protein [68], [69]. Innate immunity and adaptive immunity play a major role in hepatic stellate cell activation and propagation of liver fibrosis [70]. Anticoagulant drugs and peroxisome proliferator-activated receptor (PPAR)-γ agonists were reported to have an anti-fibrotic effect in experimental liver fibrosis, which is consistent with the enriched pathways, such as complement and coagulation cascades, and the PPAR signaling pathway [70].

**Table 2:** KEGG^a^ pathway enrichment for all genes relevant to liver fibrosis.

Pathway	Count	BH^b^
Prion diseases	11	0.003
Leukocyte transendothelial migration	19	0.008
Focal adhesion	26	0.007
Fc gamma R-mediated phagocytosis	16	0.006
Pyruvate metabolism	10	0.010
Viral myocarditis	15	0.011
Antigen processing and presentation	14	0.035
Systemic lupus erythematosus	14	0.037
Chemokine signaling pathway	21	0.035
Complement and coagulation cascades	12	0.033
Regulation of actin cytoskeleton	24	0.031
ECM^c^-receptor interaction	13	0.030
PPAR^d^ signaling pathway	12	0.029
Arginine and proline metabolism	10	0.035
Glycerolipid metabolism	9	0.035

a Kyoto Encyclopedia of Genes and Genomes

b Benjamini-Hochberg false discovery rate

c Extracellular matrix

d Peroxisome proliferator-activated receptor

**Figure 3:**
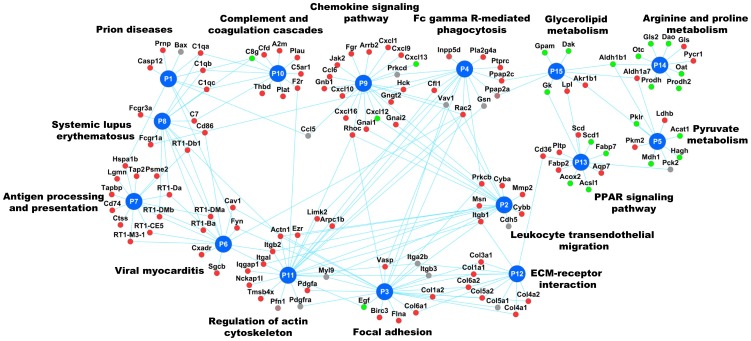
Genes that mapped to the enriched Kyoto Encyclopedia of Genes and Genomes (KEGG) pathways. The average log_2_ fold-change ratio across chemical exposures that produced periportal liver fibrosis was used as the gene expression value. Genes with average log_2_ fold-change ratios >0.6 are colored in red. Genes with average log_2_ fold-change ratios <−0.6 are colored in green. Genes whose average log_2_ fold-change ratios are between 0.6 and −0.6 are colored in grey.

We analyzed genes involved in the enriched pathways to ascertain whether we could provide new, testable hypotheses. Genes were mapped to the enriched KEGG pathways (**Figure 3**) and **Table S2** provides the complete list of these genes with pathway information, and their average log_2_ ratio in chemical exposures that produced periportal liver fibrosis. The log_2_ ratio is commonly used in microarray data analysis, and a value of 0.6 corresponds to a ∼1.5 fold-change (up-regulation) in gene expression. *Col1a1*, *Col1a2, Col4a1*, *Col4a2*, *Col5a2*, *Itgb1*, *Plat*, *Plau*, *Pdgfa*, *Ezr*, and *Msn* were up-regulated and have an average log_2_ ratio >0.6. These genes are known to be altered in liver fibrosis [4], [67], [71]. We also analyzed the gene list (**Table S2**) for potential new candidates and found genes such as *Lgmn* and *Limk2*, which were up-regulated in all four chemical exposure conditions that produced periportal liver fibrosis. These genes are potential candidates for further exploration. Next, we carried out pathway enrichment analyses using the up- and down-regulated genes separately (**Tables S3** and **S4**). The significantly up-regulated pathways were related to liver fibrosis-relevant processes, whereas the significantly down-regulated pathways were related to metabolism. The down-regulation of metabolism-related pathways could either be related to external factors, such as altered food intake, or could also be an indication of reduced liver function.

Although pathway enrichment analyses are useful and provide an overview of biological processes associated with our gene list, the method has some well-known limitations. Pathway analysis is based on curated data and is limited to the information present in the underlying knowledge database. Moreover, the pathway enrichment based on an over-representation analysis approach treats pathways as simple gene lists without accounting for network connectivity [72]. As such, pathway analysis has limited utility in identifying new molecular mediators or new pathways. Using the connectivity information or pathway topology may provide us with alternative approaches to capture the most relevant pathways associated with a disease.

### PPI network analysis

Based on the premise that proteins that are closely connected to each other in a network are more likely to be involved in similar processes, an integration of gene expression with PPI networks has been used to identify disease-specific networks [73]. Such networks have been proposed as *de novo* pathways and partly remedy the limitations of KEGG pathway analysis [25]. Consequently, we mapped the liver fibrosis-relevant genes to a high-confidence human PPI network. Out of 897 fibrosis-relevant genes, 606 mapped to the human PPI network. We extracted a liver fibrosis-relevant sub-network with 902 nodes (proteins) and 2,527 edges using KeyPathwayMiner. Out of the 606 fibrosis-relevant genes, 573 were present in this sub-network. Finally, we clustered the 902 proteins in the liver fibrosis-relevant sub-network into 11 PPI network modules. **Data S1 – S3** provides all the input and Cytoscape session files associated with PPI network analysis. **Table S5** provides the mapping of rat probe IDs to human gene IDs, and **Table S6** provides the protein membership in the PPI network modules, along with their gene expression data.

### Module characterization and potential application

We further analyzed the 11 network modules in terms of activation in chemical exposure conditions that produced periportal liver fibrosis, enrichment with known liver fibrosis-relevant genes, and enrichment of GO terms. **Table 3** shows the module activation scores 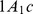 calculated using **Equation 2** for chemical exposure conditions that produced periportal liver fibrosis. Module M5 with 92 genes coding for proteins was the highest activated module with an activation score of 2.12. Next, we analyzed the enrichment of known fibrosis-relevant genes collected from the literature (**Table S7**) [3], [5], [55], [56]. **Table 3** shows the *p*-values for enrichment of known fibrosis-relevant genes in the network modules. Modules M2, M3, and M5 were enriched with liver fibrosis-relevant genes with *p*-values <0.05. Module M5 had the lowest enrichment *p*-value with six known fibrosis-associated genes coding for proteins mapped to this module: TIMP1, APOA1, CTGF, LGALS3, TGFB1, and MMP-2. CTD provides curated information on genes associated with a disease [57]. Liver fibrosis was not curated in CTD, but genes associated with liver cirrhosis, the final stage of liver fibrosis, were available. We further analyzed the enrichment of genes associated with liver cirrhosis collected from CTD (**Table S8**) and found module M5 to be the module most enriched with liver cirrhosis-related genes (**Table 3**). We also characterized the 11 modules using the GO biological process-term enrichment analysis. We found that module M5 was enriched with liver fibrosis-relevant GO terms such as *ECM organization* and *Wound healing*. **Figure S2** shows the enriched GO biological process-terms for module M5, and **Table S9** provides the entire list of enriched GO terms for all modules. Based on activation in liver fibrosis-producing conditions, enrichment of known liver fibrosis-relevant genes, and liver fibrosis-relevant GO terms, we selected module M5 as the top liver fibrosis-relevant network module identified from this analysis.

**Table 3:** Activation of network modules and enrichment of known genes relevant to liver fibrosis.

Module	Activation score in Liver fibrosis	No. of genes in module	No. of fibrosis genes (*set 1*)^a^	*p*-value (*set 1*)^a^	No. of cirrhosis genes (*set 2*)^b^	*p*-value (*set 2*)^b^
**M1**	1.47	150	1	0.24	1	1
**M2**	0.95	144	3	0.002	5	0.003
**M3**	1.67	127	2	0.02	0	1
**M4**	1.92	110	1	0.18	1	0.52
**M5**	2.12	92	6	1.28E-08	13	3.25E-14
**M6**	0.77	81	1	0.14	2	0.10
**M7**	1.09	58	-	1	1	0.32
**M8**	1.38	49	-	1	2	0.04
**M9**	0.36	46	-	1	1	0.27
**M10**	0.87	40	-	1	1	0.24
**M11**	1.38	34	-	1	1	0.20

a Liver fibrosis-relevant genes collected from literature [3], [5], [55], [56]

b Liver cirrhosis-relevant genes collected from the Comparative Toxicogenomics Database (CTD)

**Figure 4:**
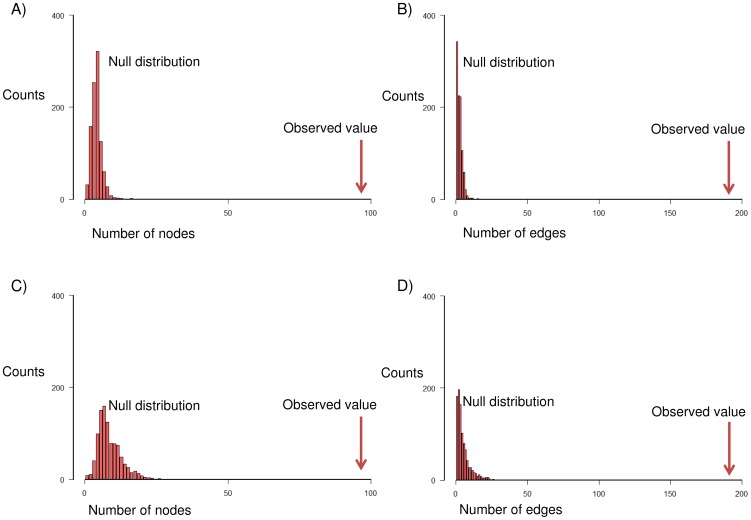
Statistical significance analysis of network module M5. A) The comparison of number of nodes in M5 to that from random sampling analysis. B) The comparison of number of edges in M5 to that from random sampling analysis. C) The comparison of number of nodes in M5 to the number present in shuffled protein-protein interaction (PPI) networks. D) The comparison of number of edges in M5 to the number present in shuffled PPI networks.

First, we ascertained whether the network module M5 could be observed by random chance. As outlined in the Materials and Methods section, we carried out two statistical significance tests based on random sampling and permutation of the network. The average node degree of the network was 12.76 and it was preserved during network shuffling. Our null hypothesis was that the number of nodes and edges in M5 could be obtained by random chance. In both tests, M5 was significantly different from random occurrence, and the probability of finding this module by random chance was essentially zero (**Figure 4**).

**Figure 5:**
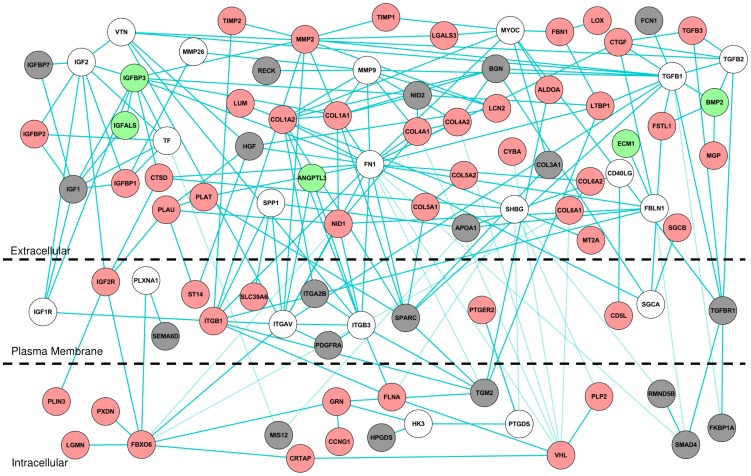
Liver fibrosis-relevant network module M5. Proteins encoded by genes with average log_2_ fold-change ratios>0.6 are colored in red. Proteins encoded by genes with average log_2_ fold-change ratios <−0.6 are colored in green. Proteins encoded by genes with average log_2_fold-change ratios between 0.6 and −0.6<−0.6 are colored in grey. Proteins without corresponding gene expression data are shown as white circles.

Second, we plotted the PPI network for the protein products encoded by the genes in module M5 (**Figure 5**). Many of the molecular interactions captured were already reported to be associated with liver fibrosis, validating the computational approach. For example, the network map shows connectivity between the genes coding for matrix metalloproteinases and the up-regulated genes encoding TIMP1, COLs and FBN1 (**Figure 5**) [4]. This network map supports a published disease mechanism where increased expression of the gene coding for TIMP1, the negative regulator of matrix metalloproteinases, leads to an increased accumulation of ECM proteins, e.g., collagens (COLs). The network module M5 included other genes encoding the proteins implicated in the pathogenesis of fibrosis (e.g., LUM, CTGF, LGALS3, LCN2, PDGFR, PLAT, and LOX) [4], [74]–[76]. Genes coding for the integrin receptors (ITG) in this network interact with ECM proteins that support their known role as mediators of pro-fibrogenic signaling of ECM proteins [77]. In addition to retrieving genes coding for proteins that are already known to be associated with liver fibrosis, this network module also retrieved some potential new candidate protein products. One such candidate is LGMN (average log_2_ ratio of 1.24), a cysteine protease that functions in ECM remodeling, but has no known associations with liver fibrosis [78]. Another candidate is the gene encoding PLIN3 (mannose-6-phosphate receptor binding protein), with an average log_2_ ratio of 1.29; it interacts with IGF2R in the M5 network. PLIN3 is known to play a role in the pathogenesis of steatosis, and is also reported to play a role in PGE2 production [79], [80].

**Figure 6:**
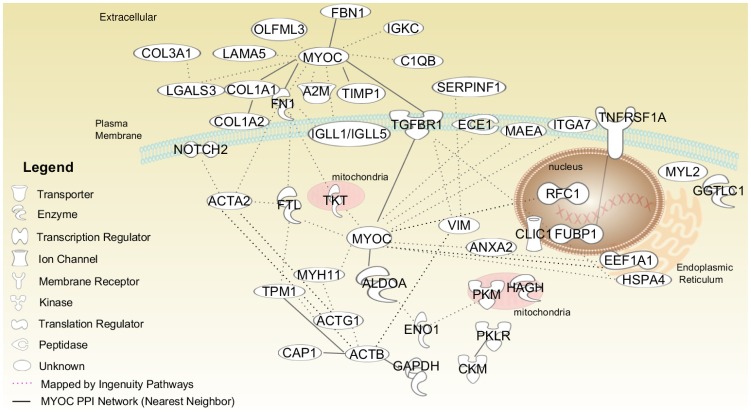
Myocilin interaction network. First neighbors of myocilin (MYOC) in the entire high-confidence human protein-protein interaction (PPI) network.

**Figure 7:**
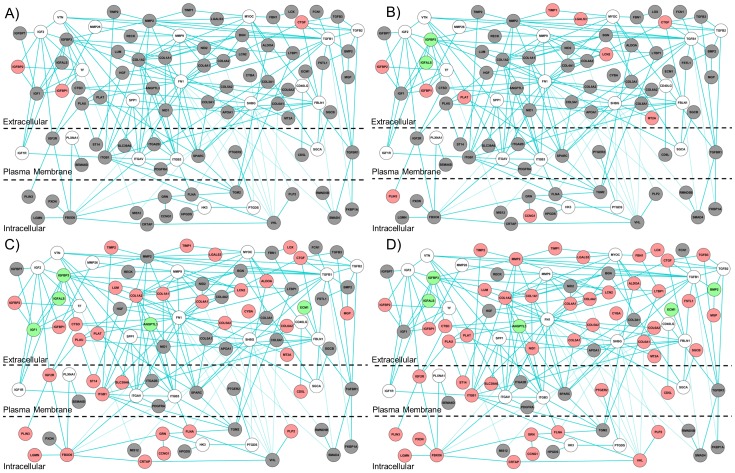
Activation of genes encoding proteins in liver fibrosis-relevant network module M5 at different time points. Genes encoding proteins with average log_2_ fold-change ratios>0.6 are colored in red. Genes encoding proteins with average log_2_ fold-change ratios <−0.6 are colored in green. Genes encoding proteins with average log_2_fold-change ratios between 0.6 and −0.6<−0.6 are colored in grey. A) Activation at 0.25-day exposure. The mapped expression profile is the average log_2_ ratio in 1-naphthyl isothiocyanate 30 mg/kg and 60 mg/kg, at 0.25-day exposure. B) Activation at 1 day of exposure. The mapped expression profile is the average log_2_ ratio in 1-naphthyl isothiocyanate 30 mg/kg and 60 mg/kg, and 4,4'-Methylenedianiline 81 mg/kg, at 1 day of exposure. C) Activation at 3 days of exposure. The mapped expression profile is the average log_2_ ratio in 1-naphthyl isothiocyanate 30 mg/kg and 60 mg/kg, and 4,4'-Methylenedianiline 81 mg/kg, at 3 days of exposure. D) Activation at >3 days of exposure. The mapped expression profile is the average log_2_ ratio across chemical exposures that produced liver fibrosis.

Third, we used the network analysis to identify proteins encoded by genes that do not change in expression, but may form PPI networks. Among the 92 proteins in M5, 30 are K-node exceptions obtained using KeyPathwayMiner, based on their connection to fibrosis-relevant proteins. In protein network analysis, the concept of guilt by association is well known [14]. If a protein is known to be associated with many proteins involved in a biological process, it can be hypothesized to play a role or to be related to the biological process associated with these proteins. For example, the genes encoding osteopontin (SPP1) and vitronectin (VTN) are known to be associated with liver fibrosis [4], [81]. These genes did not reach the fold-change threshold in our preprocessed expression dataset, but the network interactions with other fibrosis-relevant protein products that did change in expression predicted them to be fibrosis-relevant (**Figure 5**). In addition to retrieving known proteins, the network analysis also identified MYOC, a protein with no reported association with liver fibrosis. MYOC is a secreted glycoprotein involved in the pathogenesis of glaucoma [82]. MYOC interacts with many up-regulated genes in the high-confidence human PPI network, including genes that encode TIMP1, LGALS3, FBN1, COL1A2, and COL3A1 (**Figure 6**). Based on its connection with many liver fibrosis-relevant proteins, MYOC is a new testable candidate in fibrosis diagnosis and/or pathogenesis. We also analyzed the expression profile of genes in module M5 in chemical exposures that produced periportal liver fibrosis across different time periods of exposures (**Figure 7**). Only a few genes were activated (i.e., log_2_ ratio >0.6) after 0.25 day and 1 day, but at ≥3 days, most of the genes exhibited increased expression (**Figure 7**).

**Figure 8:**
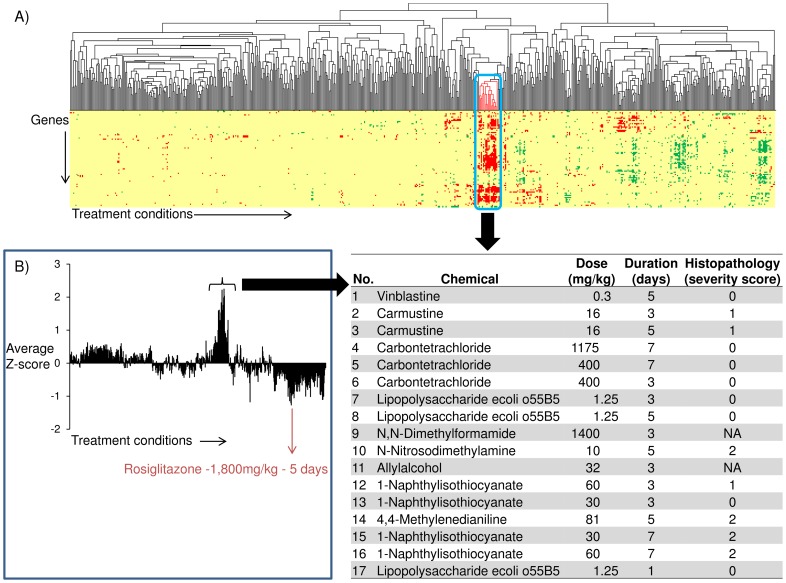
Analysis of genes in liver fibrosis-relevant network module M5. A) Hierarchical clustering of 640 chemical exposures using genes in liver fibrosis-relevant network module M5. The conditions that clustered with four liver fibrosis-producing conditions are highlighted and listed. Genes with Z-scores>2 are colored in red. Genes with Z-scores <−2 are colored in green. Genes with Z-scores between 2 and -2 are colored in yellow. NA in the table represents that histopathological data was not available for that chemical exposure condition. B) Average Z-scores across the genes in module M5 for each of the 640 chemical exposure conditions.

We performed hierarchical clustering of the 640 chemical exposure conditions present in the DrugMatrix database using the expression data for the genes in module M5. We identified a single cluster that included all four chemical exposure conditions that were initially identified with grade 2 periportal liver fibrosis (**Figure 8a**). Most of the 17 conditions in the cluster were associated with compounds that cause fibrosis. Vinblastine, carmustine, and allyl alcohol all had conditions within the drug matrix dataset that caused periportal fibrosis, but they did not meet the histopathology grade 2 threshold used for our initial analysis. Carbon tetrachloride and lipopolysaccharide exposures were present in this cluster (**Figure 8a**). Analysis of the literature shows that both carbon tetrachloride and lipopolysaccharide are well known agents that cause liver fibrosis [66]. In the DrugMatrix database, a 28-day exposure study with carbon tetrachloride showed histopathological evidence of liver fibrosis; however, gene expression data were not available for this time point. Furthermore, all but one (crotamiton-750 mg/kg exposure) of the chemicals in the DrugMatrix database that caused periportal fibrosis and had Affymetrix arrays for the liver were identified in this cluster. The data associated with the crotamiton-750 mg/kg exposure most likely represents an outlier in the underlying dataset. Thus, our analysis has the potential to generate gene sets that could predict the early onset of liver fibrosis, consistent with earlier reports that gene expression analysis of liver biopsy samples could complement histopathological data [83].

To evaluate whether network module M5 activation was specific for liver fibrosis, we plotted the average Z-scores of M5 genes in each of the 640 chemical exposure conditions (**Figure 8b**). The M5 genes were mostly activated in liver fibrosis-related chemical exposure conditions and showed very low activation in other chemical exposure conditions (**Figure 8b**). Exposure to rosiglitazone, a PPAR-γ agonist, produced an opposing effect on expression of the genes encoding the protein products in M5 (i.e., down-regulation as measured by log_2_ ratio values; **Figure S3**). This result was consistent with the evidence that PPAR-γ agonists prevented liver fibrosis in experimental models [70].

**Figure 9:**
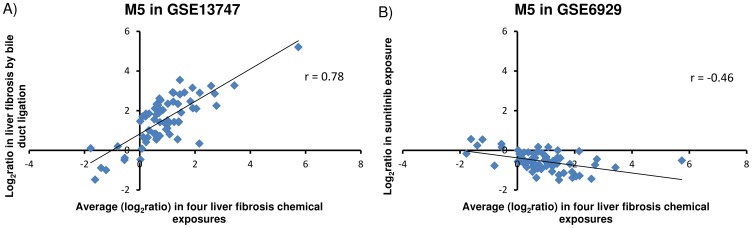
Validation with external datasets. M5 gene expression compared with external datasets. A) GSE13747 represents liver fibrosis produced by bile duct ligation. B) GSE6929 represents sunitinib (SU11248) treatment in liver cirrhosis.

Finally, we used two external gene expression datasets (GEO datasets GSE13747 and GSE6929) to evaluate the relevance of module M5 in liver fibrosis. The overlap of genes between the M5 module and the processed GSE13747 and GSE6929 datasets was 66 and 65, respectively. In the study associated with the GSE13747 dataset, liver fibrosis was induced using bile duct ligation. We observed the predicted positive correlation of gene expression data between the DEGs in this dataset and the genes in module M5 (r = 0.78, **Figure 9a**). In the GSE6929 dataset, sunitinib, a multi-kinase inhibitor, was used to treat experimental liver cirrhosis. As expected, we observed a negative correlation (r = −0.46) between log_2_ ratio gene expression data from GSE6929 and M5 (**Figure 9b**). Thus, the external dataset further supports the validity of M5 genes in liver fibrosis.

In this work, we showed the utility of integrating gene expression and PPI network analyses to understand the molecular-level details of liver fibrosis and to identify biomarker candidates. The presented computational approach has general applicability for characterizing adverse health effects. For example, our approach can be used to computationally predict toxicity pathways associated with specific diseases. Recent work has suggested that the body's response to toxic exposures and the resulting disease progression proceed through a finite set of toxicity pathways [84]. As stated earlier, the available knowledge-based pathway databases are limited in coverage and susceptible to annotation biases towards well-studied diseases. The computational approach presented in this work enabled us to partly overcome these limitations and develop *de novo* pathways linked to a specific disease. In particular, we believe that our approach could be used to further understand the molecular basis of adverse health effects such as acute kidney injury, liver cholestasis, liver steatosis, and myocardial infarction. More recently, the concept of adverse outcome pathways (AOPs) has been proposed as a novel tool in mechanism-based predictive toxicology [85]. The AOPs provide a flow-chart-like mechanistic representation of adverse health effects and consist of a molecular initiating event and a series of intermediate key events that lead to the final adverse outcome [85]. Our computational approach will be applicable to the development of novel AOPs; we believe that network modules, such as the liver fibrosis module (M5) identified in this work, could be used either as a key molecular event or used to quantify the reversibility or point of departure of key events in AOPs.

## Conclusion

We have carried out systems-level analyses of gene expression data for periportal liver fibrosis. We found that both pathway and network analyses provided insights into the molecular mechanisms of liver fibrosis. Network analyses allowed us to generate *de novo* pathways and overcome the limitations of analyses based on KEGG pathways. We identified a liver fibrosis-relevant network module that was enriched with known liver fibrosis-relevant genes predictive of liver fibrosis and validated it with external data. The systems approach used in this study allowed us to generate liver fibrosis-relevant pathways and have the potential to predict mechanism-based biomarker candidates.

## Supporting Information

Figure S1 **Dendrogram from the clustering of chemical exposure conditions that produced periportal liver fibrosis with histopathological scores >1 and their controls.**
(DOCX)

Figure S2 **Treemap view of gene ontology (GO) biological process-term enrichment for genes in the network module M5.**
(DOCX)

Figure S3 **Activation of proteins in liver fibrosis-relevant network module M5 in rosiglitazone-1,800 mg/kg, at 5 days of exposure.**
(DOCX)

Table S1 **The list of fibrosis-relevant genes from differential expression and co-expression analysis.**
(XLSX)

Table S2 **The list of genes that mapped to enriched Kyoto Encyclopedia of Genes and Genomes (KEGG) pathways and their gene expression data in chemical exposures that produced liver fibrosis.**
(XLSX)

Table S3 **The list of enriched up-regulated pathways for the liver fibrosis-relevant gene set.**
(XLSX)

Table S4 **The list of enriched down-regulated pathways for the liver fibrosis-relevant gene set.**
(XLSX)

Table S5 **Mapping of rat probe IDs to human gene IDs.**
(XLSX)

Table S6 **Protein membership in the protein-protein interaction (PPI) network modules and their gene expression data.**
(XLSX)

Table S7 **List of 28 fibrosis-relevant genes collected from literature.**
(XLSX)

Table S8 **List of 126 experimental liver cirrhosis-relevant genes collected from the Comparative Toxicogenomics Database (CTD).**
(XLSX)

Table S9 **List of enriched gene ontology (GO) terms for the protein-protein interaction (PPI) network modules, M1-M11.**
(XLSX)

Data S1 **Cytoscape session file associated with network module generation.** The Cytoscape session file includes 1) high-confidence human PPI network data, 2) the liver fibrosis-relevant parent sub-network, and 3) 11 modules obtained by clustering the liver fibrosis-relevant parent sub-network.
(ZIP)

Data S2 **KeyPathwayMiner input gene list.**
(XLSX)

Data S3 **Cytoscape session file associated with generation of the liver fibrosis-relevant parent sub-network.** The Cytoscape session file includes 1) four sub-networks obtained from KeyPathwayMiner, 2) merged nodes of four sub-networks, 3) high-confidence human PPI network data, and 4) the final liver fibrosis-relevant parent sub-network.
(ZIP)
