# Supplementary material for: Systems Level Analysis and Identification of Pathways and Networks Associated with Liver Fibrosis
Source: PLoS One. 2014 Nov 7;9(11):e112193. doi: 10.1371/journal.pone.0112193 (PMC4224449; doi:10.1371/journal.pone.0112193)
Supplement: Figure S1 — Dendrogram from the clustering of chemical exposure conditions that produced periportal liver fibrosis with histopathological scores >1 and their controls. (DOCX) [file pone.0112193.s001.docx]

**Supplementary materials**

**Systems level analysis and identification of pathways and networks associated with liver fibrosis**

Mohamed Diwan M. AbdulHameed,^1^ Gregory J. Tawa,^1^ Kamal Kumar,^1^ Danielle L. Ippolito,^2^ John A. Lewis,^2^ Jonathan D. Stallings,^2^ and Anders Wallqvist^1^

^1^Department of Defense Biotechnology High Performance Computing Software Applications Institute, Telemedicine and Advanced Technology Research Center, U.S. Army Medical Research and Materiel Command, Fort Detrick, Maryland, USA

^2^U.S. Army Center for Environmental Health Research, Fort Detrick, MD, USA


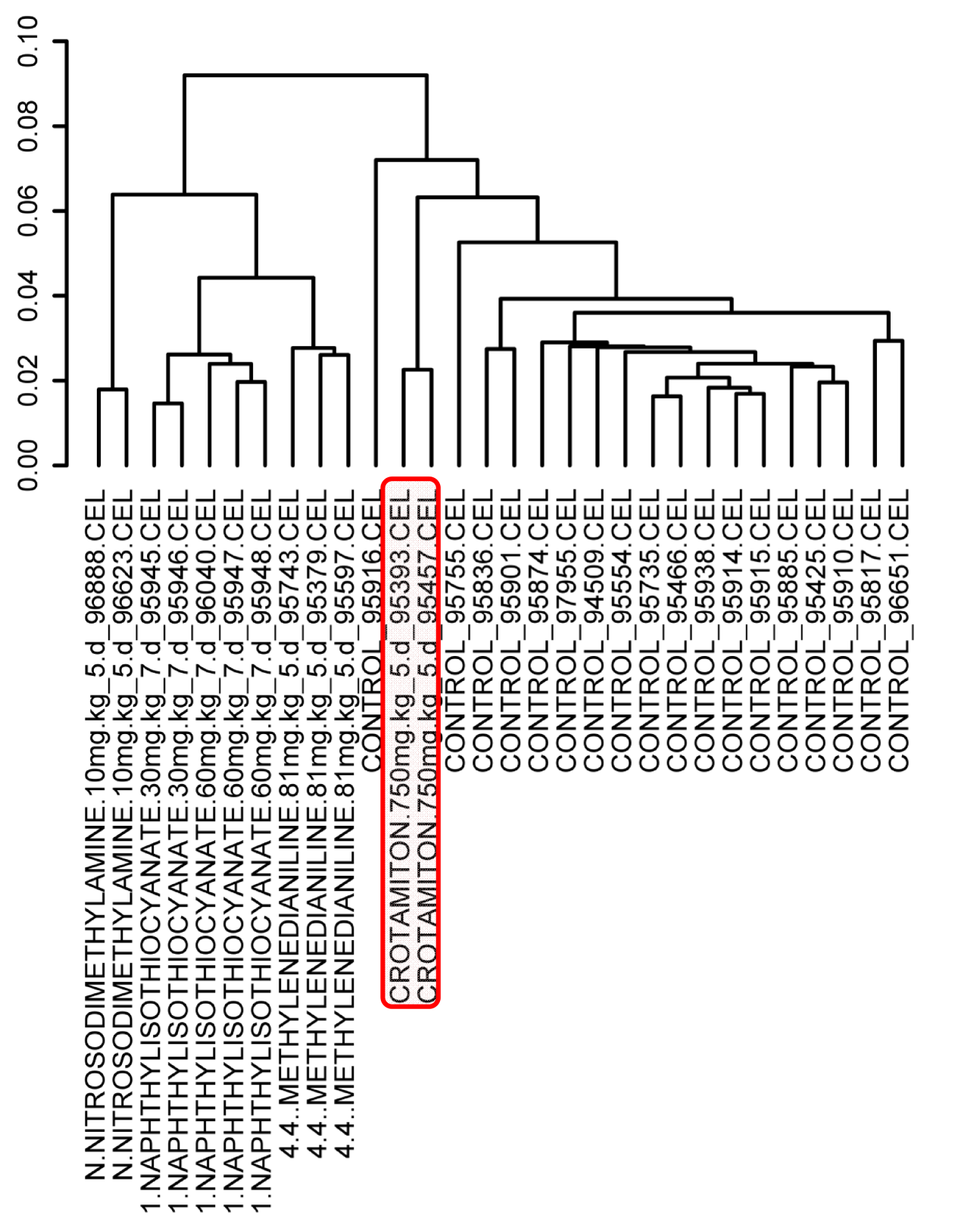


**Figure S1:** Dendrogram from the clustering of chemical exposure conditions that produced periportal liver fibrosis with histopathological score > 1 and their controls.
