# Supplementary material for: Systems Level Analysis and Identification of Pathways and Networks Associated with Liver Fibrosis
Source: PLoS One. 2014 Nov 7;9(11):e112193. doi: 10.1371/journal.pone.0112193 (PMC4224449; doi:10.1371/journal.pone.0112193)
Supplement: Figure S3 — Activation of proteins in liver fibrosis-relevant network module M5 in rosiglitazone-1,800 mg/kg, at 5 days of exposure. (DOCX) [file pone.0112193.s003.docx]

**Supplementary materials**

**Systems level analysis and identification of pathways and networks associated with liver fibrosis**

Mohamed Diwan M. AbdulHameed,^1^ Gregory J. Tawa,^1^ Kamal Kumar,^1^ Danielle L. Ippolito,^2^ John A. Lewis,^2^ Jonathan D. Stallings,^2^ and Anders Wallqvist^1^

^1^Department of Defense Biotechnology High Performance Computing Software Applications Institute, Telemedicine and Advanced Technology Research Center, U.S. Army Medical Research and Materiel Command, Fort Detrick, Maryland, USA

^2^U.S. Army Center for Environmental Health Research, Fort Detrick, MD, USA

**
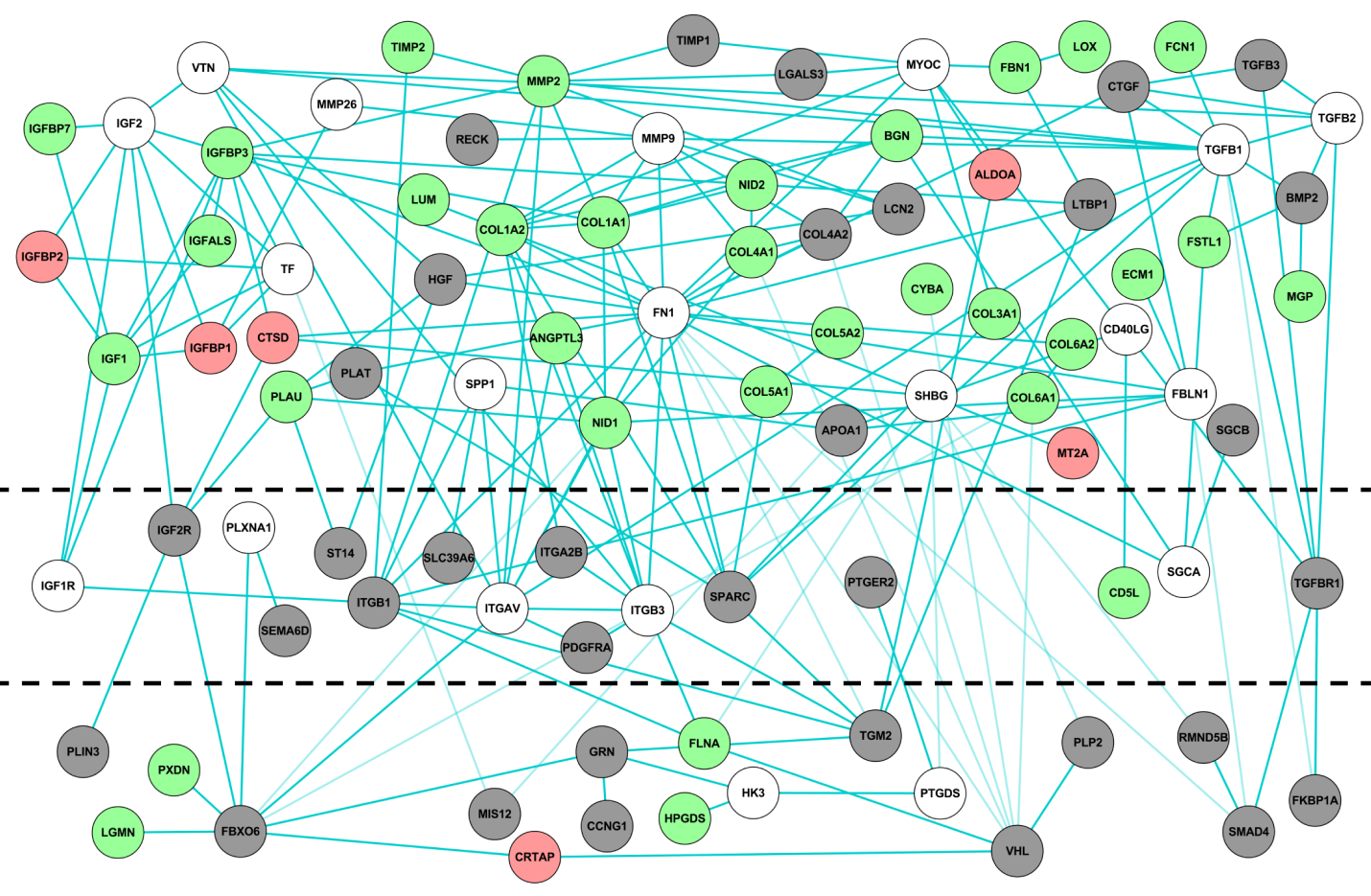
**

**Figure S3:** Activation of proteins in liver fibrosis-relevant network module M5 in rosiglitazone-1,800 mg/kg, at 5 days exposure. Proteins whose average log_2_ fold-change ratio > 0.6 are colored in red. Proteins whose average log_2_ fold-change ratio < -0.6 are colored in green. Proteins whose average log_2_ fold-change ratio is between 0.6 to -0.6 < -0.6 are colored in grey.
